# Experimental Research on ZnFe_2_O_4_@ZnCo_2_O_4_//AC@PANI Supercapacitor Energy Storage Devices for New Energy Vehicles Based on “Dual Carbon” Goals

**DOI:** 10.3390/mi17060695

**Published:** 2026-06-05

**Authors:** Yifei Wang, Yang Wang, Qing Liu, Gengchen Li, Jing Wang

**Affiliations:** 1 School of Electrical Engineering, Northeast Electric Power University, Jilin 132012, China; wyf.2000@aliyun.com (Y.W.); hmy143457@163.com (Y.W.); liuqing20011016@126.com (Q.L.); 2 School of Light Industry, Harbin University of Commerce, Harbin 150028, China

**Keywords:** core–shell structure, supercapacitor, electrode material, electrochemical performance

## Abstract

Driven by the “Dual Carbon” goals, supercapacitors have become critical energy storage devices for new energy electric vehicles. In this paper, a ZnFe_2_O_4_@ZnCo_2_O_4_ core–shell cathode was prepared by a hydrothermal method followed by high-temperature annealing, and an AC@PANI composite anode was synthesized through in situ polymerization. The materials were characterized by SEM, TEM, XRD, XPS, nitrogen adsorption–desorption and electrochemical tests. The ZnFe_2_O_4_ rod-like core provides mechanical stability, whereas the ZnCo_2_O_4_ nanosheet shell increases the specific surface area and exposes more active sites. The cathode delivers 2133 F/g at 1 A/g with 94.4% retention after 10,000 cycles. The anode reaches 398 F/g at 1 A/g. The cathode delivers 2133 F/g at 1 A/g with 94.4% retention after 10,000 cycles. The anode reaches 398 F/g at 1 A/g. The assembled ZnFe_2_O_4_@ZnCo_2_O_4_//AC@PANI hybrid supercapacitor works in a wide voltage range of 0–1.6 V. It exhibits a specific capacitance of 157 F/g at 1 A/g and a high energy density of 54.7 Wh/kg at a power density of 1600 W/kg. The device retains 91.4% of its initial capacity after 10,000 charge–discharge cycles. This study offers a promising strategy for high-performance automotive supercapacitors.

## 1. Introduction

Guided by the global “dual carbon” goals, energy structures and transportation systems are undergoing a fundamental transformation toward low-carbon, zero-carbon, and high-efficiency models. As a critical technology for carbon reduction, electric vehicles (EVs) have become central to the energy transition in the transportation sector due to their high energy efficiency and potential for zero-emission operation [1,2,3,4,5]. Driven by the rapid progress of vehicle electrification, energy storage systems need to achieve long driving mileage and endure rigorous service conditions such as frequent start-stop, hill climbing, acceleration and regenerative braking. These conditions require high instantaneous power output and input, which places strict requirements on power density, charging–discharging rate, cycle life, mechanical stability, and integration of energy storage devices [6,7,8]. Conventional lithium-ion batteries are constrained by ion diffusion kinetics and interfacial stability. Under high-rate charging and discharging, they are prone to increased polarization, capacity decay, and thermal runaway risks. Although commercial activated-carbon-based supercapacitors exhibit excellent power characteristics, their low energy density limits their ability to independently meet the long-term power demands of vehicle applications. Developing high-performance supercapacitors is essential for overcoming energy storage bottlenecks in electric vehicles. These next-generation devices must simultaneously offer high power and energy densities, exceptional stability, and a long cycle life [9,10,11].

Electrode materials and electrode architectures are key determinants of supercapacitor performance. Composite electrodes are promising for lightweight, high-power vehicle energy-storage devices because they can provide suitable electrode thickness, high active-material utilization, short ion/electron transport pathways, good mechanical flexibility, and easy integration [12,13,14,15]. However, such electrodes still face several problems. These include high interfacial resistance, insufficient active-site exposure, poor structural stability, and limited ion transport [16,17,18,19,20,21,22]. Common substrates such as nickel foam, carbon cloth, and titanium foil have good conductivity, but problems, including weak adhesion between the active material layer and substrate, easy peeling, and cracking during cycling, often lead to poor cycling stability. In addition, single-component composite electrodes face bottlenecks such as insufficient conductivity, limited active sites, and poor ion diffusion, making it hard to achieve both high specific capacitance and high rate capability [23,24,25,26,27].

Transition metal oxides, due to their abundant redox activity and high theoretical specific capacitance, have become ideal candidate materials for slurry-coated electrodes [28,29,30]. Benefiting from the synergistic effect of metal ions, spinel bimetallic oxides (e.g., ZnFe_2_O_4_, ZnCo_2_O_4_) exhibit higher conductivity, structural stability, and electrochemical activity compared with monometallic oxides. They also mitigate the drawbacks of conventional metal oxides, including low conductivity and severe volume expansion. Beyond their prominent role in electrochemical energy storage, oxide spinels have garnered extensive research interest owing to their remarkable structural flexibility, multi-functional properties, and a broad spectrum of advanced applications. Benefiting from their unique crystal chemistry—where divalent and trivalent cations occupy tetrahedral and octahedral sites—spinels serve as crucial material platforms in diverse technological fields. For instance, tailored spinel configurations exhibit outstanding optical, afterglow, thermoluminescence (TL), and optically stimulated luminescence (OSL) characteristics, making them highly attractive as advanced phosphors for optical imaging and displays [31]. Furthermore, modern paradigms are increasingly centering on the green, eco-friendly synthesis of these bimetallic systems and their hybrid composites. Such sustainable fabrication methodologies have propelled oxide spinels into multidimensional applications, ranging from environmental remediation and heterogeneous catalysis to smart electronics and automotive energy storage [32]. However, pure-phase bimetallic oxides still suffer from limited specific surface area, low ion-transport efficiency, and poor compatibility with conductive substrates, which restrict their use in high-power, long-cycle devices. Core–shell heterostructures provide an effective solution. The one-dimensional rod-shaped core offers a stable framework that buffers volume deformation during cycling, and the two-dimensional nanosheet shell increases the specific surface area and exposes more active sites. In addition, the heterojunction at the core–shell interface can optimize the electronic structure and reduce charge-transfer resistance. The as-prepared core–shell powders were further coated onto the current collector by a binder-based slurry method to ensure good mechanical contact and electrochemical stability, thereby overcoming key performance limitations of composite electrodes at both material and structural levels [33]. Although ZnFe_2_O_4_ possesses relatively low intrinsic conductivity, it is selected as the core material due to its high theoretical capacity and excellent structural stability. The 1D ZnFe_2_O_4_ nanorods not only provide a stable backbone to support the shell material but also alleviate the mechanical strain during frequent ion intercalation/de-intercalation. Furthermore, the construction of a core–shell heterostructure can effectively compensate for the conductivity limitations through the synergistic effect between the iron-based core and the highly active cobalt-based shell.

The compatibility of the anode materials also determines the overall performance of hybrid supercapacitors. Commercial activated carbon electrodes deliver stable cycling performance but low specific capacitance via electric double-layer capacitance. Polyaniline (PANI) exhibits high pseudocapacitance and good conductivity, yet suffers from agglomeration and severe volume expansion upon cycling. The AC@PANI composite anode integrates the superior stability and large specific surface area of activated carbon with the high pseudocapacitive activity of polyaniline, achieving a synergistic energy storage mechanism combining electric double-layer capacitance and pseudocapacitance. This simultaneously enhances the mechanical strength and interfacial compatibility of the film, achieving efficient matching with bimetallic oxide cathodes [34,35,36].

At present, research on composite hybrid supercapacitors based on bimetallic oxide core–shell cathodes and carbon/conductive polymer composite anodes still faces key scientific challenges: the precise mechanism for controlling the morphology of core–shell structure films remains unclear; systematic studies on the interfacial interaction patterns between the film and the conductive substrate, as well as methods for strengthening them, are lacking; strategies for potential matching between anode and cathode films, widening the voltage window, and enhancing energy density require further exploration; and the structural evolution and failure mechanisms of composite electrodes under long-cycle and high-rate conditions still need to be elucidated [37,38,39,40].

Therefore, this study targets high-power energy storage for new energy electric vehicles and focuses on improving the conductivity, structural stability, interfacial compatibility, and substrate adaptability of composite electrodes. With ZnFe_2_O_4_@ZnCo_2_O_4_ core–shell heterostructure as cathode film and AC@PANI composite as the anode film, the micro-nano structure design, composite fabrication process, substrate interface modification, and core–shell interface regulation are optimized to accelerate ion/electron transport and enhance mechanical stability.

A ZnFe_2_O_4_@ZnCo_2_O_4_//AC@PANI composite hybrid supercapacitor with a wide voltage window of 0–1.6 V is successfully assembled. The structure–performance relationship, charge storage mechanism, and cycling stability mechanism are systematically revealed. This work provides experimental evidence and theoretical support for the structural design, interfacial optimization of bimetallic oxide composite electrodes, and the practical application of high-performance automotive supercapacitors. It further facilitates transportation carbon reduction and the development of advanced energy storage technologies.

## 2. Materials and Methods

### 2.1. Materials and Characterization

#### 2.1.1. Materials

All reagents used in this experiment were of analytical grade (AR) and were used without further purification; they were used directly in the experimental preparations. The solvents used were deionized water (resistivity ≥ 18.2 MΩ·cm) and anhydrous ethanol (purity ≥ 99.7%). The specific list of reagents and their specifications are as follows: Zinc nitrate hexahydrate (Zn(NO_3_)_2_·6H_2_O), iron(III) nitrate nonahydrate (Fe(NO_3_)_3_·9H_2_O), and cobalt(II) nitrate hexahydrate (Co(NO_3_)_2_·6H_2_O) were all purchased from Shanghai Sinopharm Chemical Reagents Co., Ltd., Shanghai, China; Urea (CO(NH_2_)_2_), ammonium fluoride (NH_4_F), sodium hydroxide (NaOH), perchloric acid (HClO_4_), ammonium persulfate ((NH_4_)_2_S_2_O_8_), and aniline (ANI) were purchased from Shanghai Aladdin Biochemical Technology Co., Ltd., Shanghai, China; Activated carbon precursor (glucose) was purchased from Tianjin Comio Chemical Reagents Co., Ltd., Tianjin, China; the conductive agent Super P, the binder polyvinylidene fluoride (PVDF), and the separator (glass fiber membrane, TF4030) were purchased from Shanghai Huiping Chemical Co., Ltd., Shanghai, China.

#### 2.1.2. Characterization and Performance Testing

Various characterization techniques were employed to systematically evaluate the crystal structure, microstructure, chemical composition, and electrochemical performance of the prepared cathode and anode materials, as well as core–shell and composite materials. All tests were conducted at room temperature, with at least three parallel samples tested for each material, and the results were averaged to ensure data reliability.

The crystal structures were characterized using an X-ray diffractometer (XRD, D2 PHASER, Bruker AXS GmbH, Karlsruhe, Germany) over a 2θ range of 10–80°, with a scanning speed of 5°/min and a step size of 0.02°. Cu Kα radiation (λ = 0.15406 nm) was used at an operating voltage of 40 kV and an operating current of 40 mA. The XRD patterns were analyzed to determine the material’s crystalline phase composition, crystallinity, and grain size. Microscopic morphology and structural observation were performed using a scanning electron microscope (SEM, ZESIS SIGMA, Carl Zeiss AG, Oberkochen, Germany) and a transmission electron microscope (TEM, JEM-2100, JEOL Ltd., Tokyo, Japan). The SEM acceleration voltage was 15 kV, and the samples were gold-sputtered to improve conductivity. The TEM acceleration voltage was 200 kV, and high-resolution transmission electron microscopy (HRTEM) was used to observe lattice fringes and the interfacial characteristics of the core–shell structure. Electrochemical performance was tested using a CHI 660E electrochemical workstation (Shanghai Chenhua Instrument Co., Ltd., Shanghai, China) and a LAND battery testing system (Wuhan Landian Electronics Co., Ltd., Wuhan, China). A three-electrode system was used for single-electrode performance testing, with the prepared cathode or anode material serving as the working electrode, a platinum foil as the counter electrode, and an Hg/HgCl electrode as the reference electrode; the electrolyte was a 1 mol/L aqueous H_2_SO_4_ solution. Test methods included cyclic voltammetry (CV), galvanostatic charge–discharge (GCD), and electrochemical impedance spectroscopy (EIS).

A three-electrode system was used for single-electrode performance testing, with the prepared cathode or anode material serving as the working electrode, a platinum foil as the counter electrode, and an Hg/HgCl electrode as the reference electrode; the electrolyte was a 1 mol/L aqueous H_2_SO_4_ solution.

### 2.2. Preparation of Cathode Materials

#### 2.2.1. Preparation of ZnFe_2_O_4_ Materials

ZnFe_2_O_4_ nanorods were prepared using a hydrothermal method combined with a high-temperature annealing process, as described below: First, based on a 1:2 molar ratio of Zn^2+^ to Fe^3+^, 0.297 g of Zn(NO_3_)_2_·6H_2_O and 1.011 g of Fe(NO_3_)_3_·9H_2_O were accurately weighed and placed in a beaker. Sixty milliliters of deionized water were added, and the mixture was magnetically stirred for 30 min until completely dissolved, yielding a homogeneous mixed salt solution. Next, 0.30 g of urea and 0.093 g of NH_4_F were slowly added to the mixed solution, and magnetic stirring was continued for 1 h until both reagents were completely dissolved. At this point, the solution appears pale yellow and transparent. Urea acts as a reducing agent and homogeneous precipitant, while NH_4_F serves as a morphology control agent, effectively regulating the particle size and dispersion of the product. The mixed solution was transferred to a 100 mL PTFE-lined stainless steel autoclave, sealed, and placed in a forced-air drying oven. The temperature was maintained at 120 °C for 6 h, and the mixture was then allowed to cool naturally to room temperature. The reactor was opened, and the precipitate was collected and centrifugally washed five times alternately with deionized water and anhydrous ethanol. Each centrifugation was performed at 8000 rpm for 5 min to remove unreacted reagents and impurities. Subsequently, the precipitate was dried in a vacuum oven at 80 °C for 12 h to obtain the ZnFe_2_O_4_ precursor powder. The dried precursor powder was placed in a porcelain boat and transferred to a muffle furnace for high-temperature annealing in air at a heating rate of 5 °C/min. After the temperature reached 400 °C, it was maintained for 2 h and then allowed to cool naturally to room temperature, yielding black ZnFe_2_O_4_ nanoparticle powder, which was sealed and stored for future use.

#### 2.2.2. Preparation of ZnCo_2_O_4_ Materials

Zn(NO_3_)_2_·_6_H_2_O (0.297 g) and Co(NO_3_)_2_·_6_H_2_O (0.749 g) were weighed according to a 1:2 molar ratio of Zn^2+^ to Co^2+^, dispersed in 60 mL of deionized water, and magnetically stirred for 30 min to obtain a homogeneous mixed salt solution. Then, 0.30 g of urea and 0.093 g of NH_4_F were added, and magnetic stirring was continued for 1 h to obtain a homogeneous, pale pink, transparent solution. Transfer the mixture to a 100 mL stainless steel autoclave lined with polytetrafluoroethylene (PTFE). The reactor was sealed and maintained in a forced-air drying oven at 120 °C for 6 h. After the reaction, the mixture was allowed to cool naturally to room temperature. The reaction product was collected and centrifugally washed five times alternately with deionized water and anhydrous ethanol (8000 rpm, 5 min per wash) to remove impurities. After washing, the product was dried in a vacuum oven at 80 °C for 12 h, yielding ZnCo_2_O_4_ precursor powder. The precursor powder was placed in a porcelain boat and placed in a muffle furnace for annealing under an air atmosphere. The heating rate was 5 °C/min; after reaching 400 °C, the temperature was maintained for 2 h, followed by natural cooling to room temperature, yielding black ZnCo_2_O_4_ nanopowder, which was sealed and stored for future use. As a typical spinel-type transition metal oxide, ZnCo_2_O_4_ exhibits excellent electrical conductivity and pseudocapacitive properties; optimization of its preparation process can effectively enhance the material’s electrochemical activity.

#### 2.2.3. Preparation of ZnFe_2_O_4_@ZnCo_2_O_4_ Core–Shell Structure

**Figure 1:**
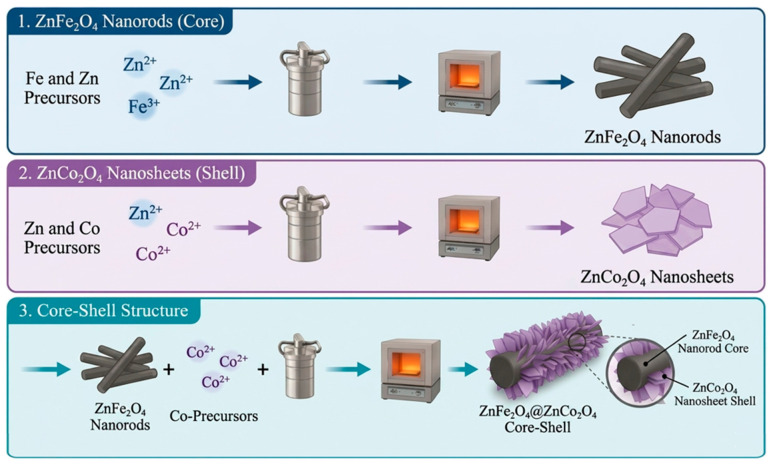
Schematic representation of the step-by-step synthesis of ZnFe_2_O_4_@ZnCo_2_O_4_ core–shell structured materials (Original illustration personally created by the authors).

First, 0.1 g of ZnFe_2_O_4_ nanopowder was dispersed in 60 mL of deionized water and sonicated for 30 min to obtain a uniform ZnFe_2_O_4_ suspension, which provided a well-dispersed foundation for uniform shell growth. Next, 0.297 g of Zn(NO_3_)_2_·_6_H_2_O and 0.749 g of Co(NO_3_)_2_·_6_H_2_O were added to the suspension according to a 1:2 molar ratio of Zn^2+^ to Co^2+^, followed by magnetic stirring for 30 min to disperse the metal ions uniformly. Next, 0.30 g of urea and 0.093 g of NH_4_F were added, and magnetic stirring was continued for 1 h to yield a well-dispersed homogeneous suspension. This suspension was transferred to a 100 mL stainless steel autoclave lined with polytetrafluoroethylene (PTFE). After sealing, the reactor was placed in a forced-air drying oven at 120 °C for a 6 h isothermal reaction, followed by natural cooling to room temperature. The reaction product was collected and centrifugally washed five times alternately with deionized water and anhydrous ethanol (8000 rpm, 5 min per wash) to remove unreacted reagents and free ZnCo_2_O_4_ particles. It was then dried in a vacuum oven at 80 °C for 12 h, yielding the ZnFe_2_O_4_@ZnCo_2_O_4_ core–shell structure precursor. The precursor was placed in a porcelain boat and loaded into a muffle furnace. Under an air atmosphere, the temperature was raised at a rate of 5 °C/min to 400 °C, held at this temperature for 2 h, and then allowed to cool naturally to room temperature, yielding a black ZnFe_2_O_4_@ZnCo_2_O_4_ core–shell composite material. The detailed synthetic route for the as-prepared ZnFe_2_O_4_@ZnCo_2_O_4_ core–shell composite is schematically displayed in Figure 1.

### 2.3. Preparation of Anode Materials

#### 2.3.1. Preparation of AC Materials

Activated carbon (AC) material for supercapacitor anodes was prepared via a template method combined with a chemical activation process. First, 5 g of glucose and 2 g of nano-SiO_2_ were accurately weighed and dispersed in 50 mL of deionized water. The mixture was magnetically stirred for 30 min to obtain a homogeneous solution, which was then transferred to a 100 mL autoclave lined with polytetrafluoroethylene (PTFE). The hydrothermal reaction was carried out at 180 °C for 12 h to prepare the carbon-based precursor. After the reaction, the mixture was allowed to cool naturally to room temperature. The as-prepared hydrothermal product was collected, washed with deionized water until the filtrate reached neutrality, and vacuum-dried at 80 °C for 12 h to obtain a carbon-template composite. The composite was placed in a porcelain boat and transferred to a muffle furnace. Under a nitrogen atmosphere, the temperature was raised at a rate of 5 °C/min to 800 °C, where it was held for 2 h for carbonization. After cooling to room temperature, the product was immersed in a 1 mol/L HF solution for 24 h to remove the nano-SiO_2_ template, then washed with deionized water until neutral and vacuum-dried at 80 °C for 12 h. The dried carbon material was blended with KOH at a mass ratio of 1:4, followed by activation in a muffle furnace at 800 °C for 2 h under nitrogen. The heating rate was set at 5 °C/min. After natural cooling to room temperature, the product was rinsed with deionized water to neutrality and vacuum-dried at 80 °C for 12 h to yield activated carbon (AC) powder, which was sealed for subsequent use.

#### 2.3.2. Preparation of PANI Material

Polyaniline (PANI) was synthesized via oxidative chemical polymerization using aniline (ANI) as the monomer, ammonium persulfate as the oxidizing agent, and perchloric acid as the proton acid dopant. The procedure was as follows. Under an ice-water bath (0–5 °C), 0.01 mol of aniline monomer was slowly added to 100 mL of 1 mol/L perchloric acid solution, followed by magnetic stirring for 30 min to obtain a homogeneous acidic aniline solution. The ice-water bath effectively controls the polymerization reaction rate, preventing vigorous reaction activity that could lead to product agglomeration. In a separate 100 mL portion of 1 mol/L perchloric acid solution, 0.01 mol of ammonium persulfate was dissolved to prepare the oxidant solution. This solution was then added dropwise to the aniline acid solution at a rate of 1 drop per second, and the reaction was stirred in the ice-water bath for another 6 h. At this point, the solution gradually turns dark green, and PANI precipitate forms. After the reaction, the precipitate was collected by centrifugation (8000 rpm, 5 min) and washed five times alternately with deionized water and anhydrous ethanol to remove unreacted aniline monomer, oxidant, and impurities until the filtrate became colorless and transparent. The precipitate was dried in a vacuum oven at 60 °C for 48 h to obtain dark-green PANI powder, which was sealed and stored for subsequent characterization and electrochemical testing.

#### 2.3.3. Preparation of AC@PANI Material

AC@PANI composites were prepared using an in situ polymerization method. First, 0.2 g of AC powder was accurately weighed and dispersed in 100 mL of 1 mol/L perchloric acid solution. The mixture was sonicated for 30 min to obtain a uniform AC suspension, ensuring that the AC particles were fully dispersed to provide sufficient active sites for the in situ polymerization of PANI.

**Figure 2:**
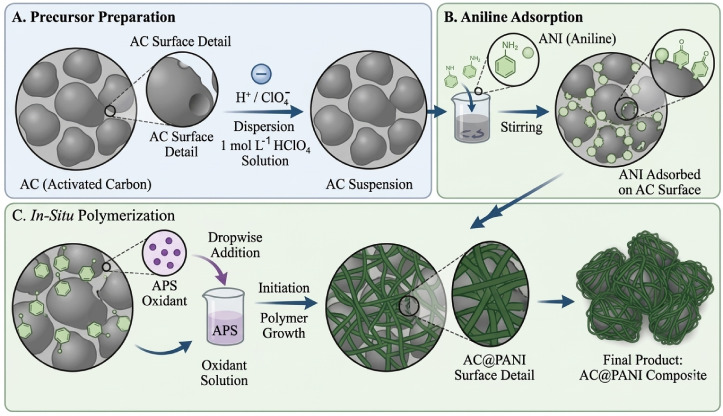
Schematic representation of the step-by-step synthesis of AC@PANI core–shell structured materials. (Original illustration personally created by the authors).

Under an ice-water bath (0–5 °C), 0.01 mol of aniline monomer was introduced into the AC suspension with continuous magnetic stirring for 30 min to achieve sufficient monomer adsorption on the AC surface. Thereafter, a preconfigured ammonium persulfate-perchloric acid solution (0.01 mol ammonium persulfate dissolved in 100 mL of 1 mol/L perchloric acid) was slowly dropwise added at a rate of one drop per second. Upon completion of the addition, stirring was continued in the ice-water bath for 6 h to achieve in situ polymerization of PANI on the AC surface. After the reaction, the product was collected by centrifugation (8000 rpm, 5 min) and washed five times alternately with deionized water and anhydrous ethanol to remove unreacted monomers, oxidants, and free PANI until the washing solution became colorless and transparent. The product was dried in a vacuum oven at 60 °C for 48 h to obtain AC@PANI composite powder, which was sealed and stored for future use. The synthetic procedure of AC@PANI core-shell composite is schematically illustrated in Figure 2.

### 2.4. Assembly of Supercapacitors

In this experiment, a button-type supercapacitor (CR2032 type) was assembled using the prepared ZnFe_2_O_4_@ZnCo_2_O_4_ core–shell material as the cathode, AC@PANI composite as the anode, a 1 mol/L H_2_SO_4_ aqueous solution as the electrolyte, and a glass fiber membrane as the separator.

The electrode preparation process is as follows: Weigh the cathode active material (ZnFe_2_O_4_@ZnCo_2_O_4_) and anode active material (AC@PANI) separately, then mix each with Super P conductive carbon black and PTFE emulsion in a mass ratio of 8:1:1. Add an appropriate amount of anhydrous ethanol and grind for 10 min to form a uniform paste-like electrode slurry. The slurry was evenly coated onto a nickel foam current collector (1 cm × 1 cm), placed in a vacuum oven at 60 °C for 24 h to remove the solvent, and then pressed at 10 MPa for 5 min to obtain the cathode and anode sheets. The mass of the active materials on the positive and negative electrodes was balanced according to the charge balance principle (Q+ = Q−). Based on the formula *m*^+^/*m*^−^ = *C*^−^ × Δ*V*^−^/*C*^+^ × Δ*V*^+^, and considering the specific capacitances of the cathode (2133 F/g) and anode (398 F/g), the actual mass ratio of positive to negative active materials in the assembled device was controlled at approximately 0.31. In the actual device assembly, the mass loading of the cathode active material was approximately 1.1 mg, while that of the anode was approximately 3.5 mg.

For supercapacitor assembly, the positive terminal cap of a CR2032 button-cell casing was placed in a glove box. The positive electrode sheet and glass-fiber separator were then inserted sequentially, and 3–4 drops of 1 mol/L H_2_SO_4_ electrolyte were added to fully wet the separator. Finally, the negative electrode sheet, spacer, and spring clip were inserted. The device was sealed with a negative cap and mechanically pressed to finish the assembly of coin-type supercapacitors. The assembled cells were aged at room temperature for 12 h to guarantee adequate electrolyte infiltration and favorable electrode-electrolyte interfacial contact. Electrochemical performance testing was then performed. The assembled supercapacitors were tested using a two-electrode system. The CV test covered a voltage range of 0–1.6 V, the GCD test used a current density of 0.5–10 A/g, and the EIS test spanned a frequency range of 0.01 Hz–100 kHz to evaluate the overall energy storage performance of the supercapacitors. The electrochemical performance of the assembled asymmetric supercapacitor was evaluated using a two-electrode system. The specific capacitance (*C_s_*, F/g), energy density (*E*, Wh/kg), and power density (*P*, W/kg) of the ASC device were all calculated based on the total active mass of both the cathode and anode materials. The specific capacitance was calculated using *C* = *I* × Δt/(*M* × Δ*V*), where *M* (*M* = *m*^+^ + *m*^−^) represents the total mass loading of the active materials on both electrodes.

For the three-electrode system (single electrode)*:*

Cs=IΔt/mΔV

For the two-electrode system (ASC device):

Cdevice=IΔt/MΔV

$$E=0.5C\mathrm{device}\times \Delta V^{2}$$

P=3600EΔt

Here, *I*(*A*) is the discharge current, Δ*t* (s) is the discharge time, and Δ*V* (*V*) is the operating potential window during discharge after excluding the voltage drop. The term m (g) denotes the mass of active material on a single working electrode in the three-electrode tests, whereas *M*(g) is the total active-material mass of the positive and negative electrodes (*M* = *m*^+^ + *m*^−^) used for device-level calculations of *C*_device_, *E*, and *P*. *C_s_* and *C*_device_ (F/g) represent the specific capacitances of the electrode and the assembled device, respectively.

## 3. Results and Discussion

**Figure 3:**
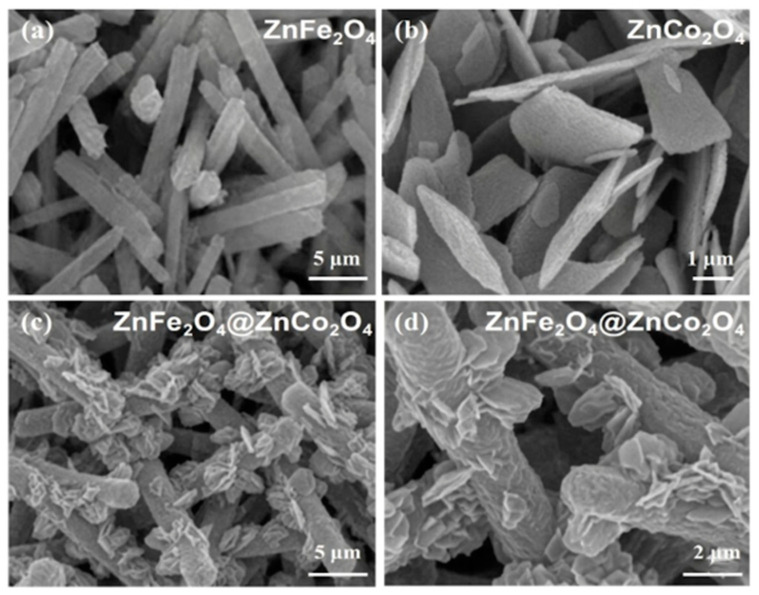
Microscopic morphology characterization of different electrode materials: (**a**) pure-phase ZnFe_2_O_4_ one-dimensional rod-like structure; (**b**) pure-phase ZnCo_2_O_4_ nanosheets; (**c**,**d**) low- and high-magnification SEM images of the ZnFe_2_O_4_@ZnCo_2_O_4_ composite material (ZnCo_2_O_4_ nanosheets grown on the rod-like ZnFe_2_O_4_ structure).

To investigate the evolution of the material’s microstructure, we systematically characterized the structural features of the products at different stages using scanning electron microscopy (SEM). The results are shown in Figure 3. Figure 3a shows the SEM image of the pure-phase ZnFe_2_O_4_ precursor. The sample exhibits a uniform one-dimensional rod-like structure overall, with a smooth surface and distinct contours. The rods have an average length of 10–20 μm and a diameter of 1–2 μm. The thickness of the final composite electrode coating on the nickel foam current collector was controlled at approximately 100–120 μm to ensure both high mass loading and efficient ion transport. This anisotropic structure with a controllable aspect ratio provides an ideal skeletal framework for the subsequent construction of heterostructures. In Figure 3b, the pure ZnCo_2_O_4_ sample exhibits a two-dimensional, flake-like aggregation morphology, resulting from the disordered stacking of nanosheets to form irregular aggregates. Such randomly stacked nanosheets are prone to structural collapse and hindered ion transport during repeated charge–discharge cycling.

In contrast, the ZnFe_2_O_4_@ZnCo_2_O_4_ composite constructed by in situ growth exhibits a markedly different microstructure (Figure 3c,d). Low-magnification SEM images (Figure 3c) clearly show that the rod-like ZnFe_2_O_4_ scaffold is well preserved after the growth process. The ZnCo_2_O_4_ nanosheet shell presents a hierarchically porous structure, with nanosheets of approximately 20 nm in thickness uniformly and densely anchored on the substrate. The ZnFe_2_O_4_ core acts as a robust conductive skeleton and contributes dominant surface-dominated electric double-layer capacitance (EDLC). When coupled with the high-pseudocapacitance ZnCo_2_O_4_ shell, the heterostructure optimizes the balance between fast ion adsorption and deep-level redox reactions, resulting in the observed superior performance compared to single-component electrodes. High-magnification SEM images (Figure 3d) further reveal that the ZnCo_2_O_4_ nanosheets grow vertically on the rod-like substrate rather than being randomly attached, forming a hierarchical core–shell array structure. This vertically aligned morphology provides an open framework that facilitates electrolyte penetration. First, the one-dimensional ZnFe_2_O_4_ core improves the mechanical stability of the overall structure and effectively alleviates volume expansion during cycling. Second, the two-dimensional ZnCo_2_O_4_ nanosheet shell greatly increases the specific surface area and exposes more electrochemically active sites. Third, the heterojunction formed at the core–shell interface can regulate the electronic structure and facilitate ion diffusion kinetics. These structural features collectively contribute to the improved rate capability and cycling stability of the supercapacitor.

**Figure 4:**
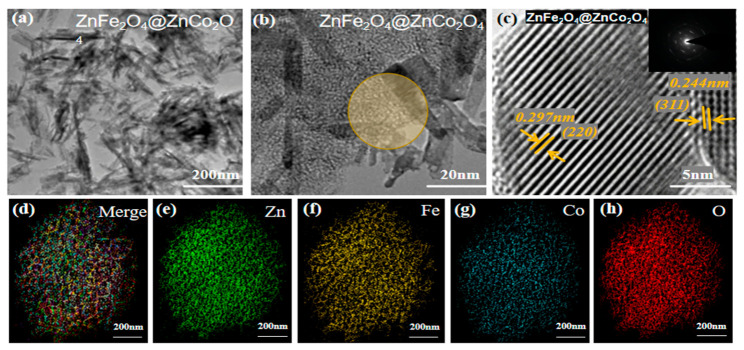
Microstructural characterization of the ZnFe_2_O_4_@ZnCo_2_O_4_ composite: (**a**,**b**) TEM images at different magnifications; (**c**) HRTEM image and corresponding SAED pattern; (**d**–**h**) EDS elemental mapping images of Zn, Fe, Co, and O.

To further elucidate the fine structure and elemental distribution of the ZnFe_2_O_4_@ZnCo_2_O_4_ composite, we performed characterization using transmission electron microscopy (TEM), high-resolution transmission electron microscopy (HRTEM), and energy-dispersive X-ray spectroscopy (EDS) with area scanning. The results are shown in Figure 4. Low-magnification TEM images (Figure 4a,b) clearly show the core–shell multiscale structure: a layer of ZnCo_2_O_4_ nanosheets uniformly encapsulates the surface of the one-dimensional rod-shaped ZnFe_2_O_4_ core. The interface between the core and shell is distinct and intimately bonded, with no obvious phase separation. This in situ-grown heterogeneous interface effectively reduces interfacial resistance and promotes charge transfer. To further confirm the crystalline structure of the ZnFe_2_O_4_@ZnCo_2_O_4_ composite, we re-characterized the sample by high-resolution transmission electron microscopy (HRTEM), as shown in Figure 4c. The newly acquired HRTEM image presents clear, continuous, and well-defined lattice fringes. The interplanar spacing of 0.297 nm corresponds to the (220) crystal plane of spinel ZnFe_2_O_4_, while the interplanar spacing of 0.244 nm matches the (311) crystal plane of spinel ZnCo_2_O_4_. The corresponding selected-area electron diffraction (SAED) pattern (inset of Figure 4c) displays distinct polycrystalline diffraction rings, further confirming the coexistence of the ZnFe_2_O_4_ and ZnCo_2_O_4_ phases and the high crystallinity of the composite. To verify the uniform distribution of elements, we performed EDS elemental mapping analysis (Figure 4d–h). The composite elemental image in Figure 4d and the distribution maps of Zn, Fe, Co, and O in Figure 4e–h show that the four elements—Zn, Fe, Co, and O—are uniformly distributed throughout the entire core–shell structure, with no obvious elemental segregation or local enrichment. Among these, Fe is primarily concentrated in the inner core region, while Co is more densely distributed in the outer shell region. This aligns closely with the core–shell structural characteristics of ZnFe_2_O_4_@ZnCo_2_O_4_, further confirming the successful construction of the heterostructure. This uniform elemental distribution, combined with the tight core–shell interface, not only facilitates rapid electron transport but also maintains structural integrity during charging and discharging, thereby providing a strong guarantee for the rate capability and cycling stability of supercapacitor electrode materials.

**Figure 5:**
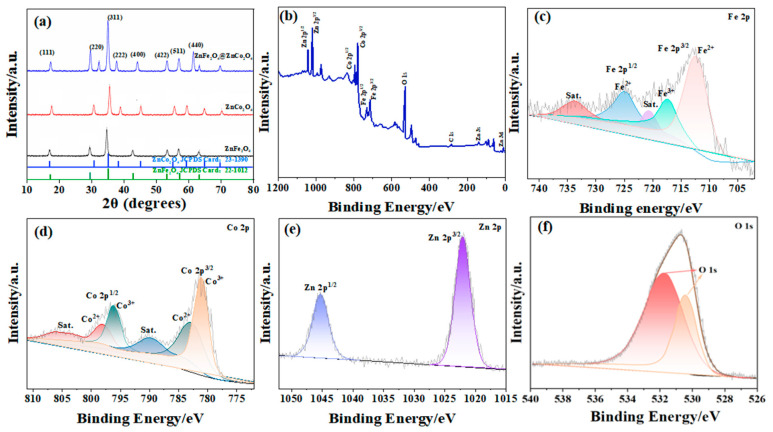
Characterization of the phase and surface chemical states of different samples: (**a**) XRD spectra of ZnFe_2_O_4_, ZnCo_2_O_4_, and ZnFe_2_O_4_@ZnCo_2_O_4_; (**b**) Full XPS spectrum of ZnFe_2_O_4_@ZnCo_2_O_4_; (**c**–**f**) High-resolution XPS spectra of ZnFe_2_O_4_@ZnCo_2_O_4_, corresponding to the Fe 2p, Co 2p, Zn 2p, and O 1s orbitals, respectively.

To systematically investigate the phase structure and surface chemical state of the ZnFe_2_O_4_@ZnCo_2_O_4_ composite, we performed X-ray diffraction (XRD) and X-ray photoelectron spectroscopy (XPS) characterization. The results are presented in Figure 5. As shown in Figure 5a, the characteristic diffraction peaks of pure ZnFe_2_O_4_ are in good agreement with the standard JCPDS card No. 22-1012, corresponding to the typical crystal planes of the spinel structure. In addition, the diffraction peaks of pure ZnCo_2_O_4_ match well with the standard JCPDS card No. 23-1390, confirming the successful formation of both spinel phases. The XRD pattern of the ZnFe_2_O_4_@ZnCo_2_O_4_ composite exhibits characteristic diffraction peaks of both ZnFe_2_O_4_ and ZnCo_2_O_4_ simultaneously, with no obvious impurity peaks. This indicates that no impurities were introduced during the in situ growth process, that the crystal structures of both spinel phases were fully preserved, and that the composite possesses high crystallinity. To further understand the microstructural evolution, the lattice parameters and crystallite sizes were calculated. The average crystalite size (*D*) was estimated using the Scherrer formula (*D = kλ/β cos θ*). Furthermore, the dislocation density (*δ = 1/D*^2^) and microstrain (*ε* = *β*/4 *tan θ*)were determined to assess the lattice defects. As shown in Table 1, the composite shows a slight change in lattice parameters compared to pure ZnFe_2_O_4_, which can be attributed to the interfacial strain between the core and the ZnCo_2_O_4_ shell. The increase in microstrain in the composite further confirms the tight coupling and lattice mismatch at the heterojunction interface. The XPS full spectrum (Figure 5b) further confirmed the coexistence of the four elements Zn, Fe, Co, and O in the composite, which is consistent with the EDS elemental mapping results. High-resolution XPS spectra provide detailed information about the valence states of the elements. As shown in Figure 5c, the Fe 2p spectrum can be deconvoluted into Fe^2+^ and Fe^3+^ components accompanied by satellite peaks, indicating the coexistence of mixed Fe valence states in ZnFe_2_O_4_. Such mixed valence states can provide additional redox-active sites. Similarly, the Co 2p spectrum shown in Figure 5d consists of Co^2+^ and Co^3+^ species together with obvious satellite peaks, which are beneficial for charge transfer during electrochemical reactions. In the Zn 2p spectrum (Figure 5e), the characteristic peaks located at approximately 1021.5 and 1044.6 eV correspond to Zn 2p_3/2_ and Zn 2p_1/2_, respectively, confirming the presence of Zn^2+^. Moreover, the O 1s spectrum in Figure 5f can be divided into lattice oxygen and surface-adsorbed oxygen/hydroxyl oxygen. The adsorbed oxygen species may provide additional pathways for ion diffusion and facilitate surface redox reactions. In summary, the XRD and XPS results jointly confirm the successful fabrication of the ZnFe_2_O_4_@ZnCo_2_O_4_ heterostructure. The crystal structures of the two spinel phases are intact, and the coexistence of multivalent Fe and Co species with abundant reactive oxygen species on the surface provides a solid structural and chemical foundation for the excellent electrochemical performance of the composite material.

**Figure 6:**
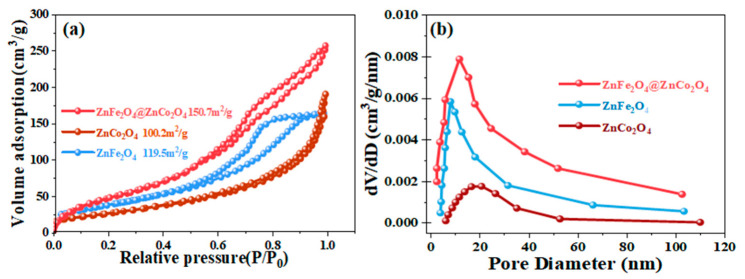
Characterization of pore structures in different samples: (**a**) N_2_ adsorption–desorption isotherms and corresponding specific surface areas; (**b**) Pore size distribution curves.

To investigate the effects of the pore structure and specific surface area of the materials on their electrochemical performance, we conducted nitrogen adsorption and desorption tests on ZnFe_2_O_4_, ZnCo_2_O_4_, and ZnFe_2_O_4_@ZnCo_2_O_4_ samples. The results are shown in Figure 6. As shown in the nitrogen adsorption–desorption isotherms in Figure 6a, all three samples exhibit typical Type IV isotherms accompanied by H3-type hysteresis loops, indicating the presence of mesoporous structures within the materials. The pore system primarily consists of open-pore channels formed by slits or stacking gaps, which effectively facilitate electrolyte infiltration and accelerate ion transport kinetics. The calculated specific surface area of the ZnFe_2_O_4_@ZnCo_2_O_4_ composite is 150.7 m^2^/g, which is significantly higher than that of pure ZnFe_2_O_4_ (119.5 m^2^/g) and ZnCo_2_O_4_ (100.2 m^2^/g). This significant enhancement is largely ascribed to the introduction of ZnCo_2_O_4_ nanosheet arrays within the core–shell hierarchical structure, which not only increases the material’s surface exposure but also creates a rich mesoporous network. Further pore size distribution curves (Figure 6b) indicate that the pore sizes of the composite are primarily concentrated in the mesoporous range of 2–20 nm, and the pore volume is significantly higher than that of the two pure-phase materials. The large specific surface area and abundant mesoporous structure exert a synergistic effect, which provides abundant active sites for electrochemical reactions, shortens ion diffusion pathways, and reduces charge transfer resistance. Such structural advantages are beneficial to improving the rate performance and cycling stability of supercapacitors.

**Figure 7:**
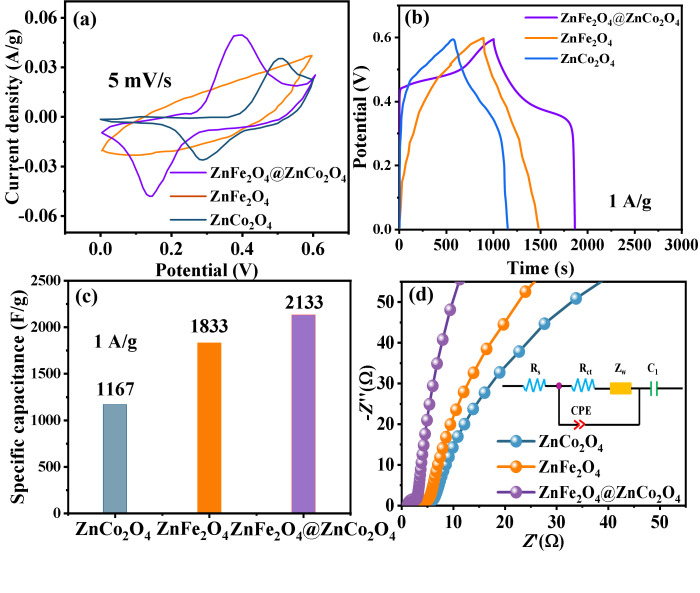
Comparison of electrochemical performance of different electrode materials: (**a**) CV curve at a scanning rate of 5 mV/s; (**b**) GCD curve at a current density of 1 A/g; (**c**) Comparison of specific capacitance at 1 A/g; (**d**) Nyquist curve of electrochemical impedance spectroscopy (EIS).

To systematically evaluate the electrochemical performance of the ZnFe_2_O_4_@ZnCo_2_O_4_ core–shell heterostructure as a supercapacitor cathode material, cyclic voltammetry (CV), galvanostatic charge–discharge (GCD), and electrochemical impedance spectroscopy (EIS) measurements were carried out. Pure ZnFe_2_O_4_ and ZnCo_2_O_4_ electrodes were also investigated for comparison. The results are shown in Figure 7. Figure 7a shows the CV curves of the three electrodes at a scan rate of 5 mV/s. All curves display a pair of well-defined redox peaks, confirming the pseudocapacitive charge-storage mechanism typical of spinel oxides. The CV curve area of the ZnFe_2_O_4_@ZnCo_2_O_4_ composite is much larger than that of the two pure-phase materials, and its redox peaks are more symmetric, indicating higher specific capacity and better reaction reversibility. This phenomenon stems from the synergistic effects at the heterojunction within the core–shell structure, which not only maintains the high theoretical specific capacitance of the ZnFe_2_O_4_ core but also provides abundant redox active sites via the ZnCo_2_O_4_ shell, thereby improving the overall reaction kinetics. The electrochemical performance of the ZnFe_2_O_4_ core was further analyzed. It is noteworthy that ZnFe_2_O_4_ exhibits a composite charge-storage mechanism. The quasi-rectangular shape of the CV curves at high scan rates suggests a non-negligible electric double-layer capacitance (EDLC) contribution, likely due to the large effective surface area of the 1D nanorod structure. However, the distinct redox peaks and the total specific capacitance values indicate that faradaic pseudocapacitance remains a major contributor. This hybrid mechanism allows the material to maintain high capacitance across a wide range of current densities. Figure 7b shows the GCD curves of the three electrodes at a current density of 1 A/g. All curves show nearly symmetric charge–discharge platforms, demonstrating good reversibility of the pseudocapacitive energy storage. The ZnFe_2_O_4_@ZnCo_2_O_4_ electrode delivers a much longer discharge duration than pure ZnFe_2_O_4_ and ZnCo_2_O_4_, indicative of its superior charge storage capability. As shown in the specific capacitance histogram in Figure 7c, the ZnFe_2_O_4_@ ZnCo_2_O_4_ electrode delivers a high specific capacitance of 2133 F/g at a current density of 1 A/g. This value is significantly higher than those of pure ZnFe_2_O_4_ (1833 F/g) and ZnCo_2_O_4_ (1167 F/g), indicating a remarkable improvement compared with the single-component materials. The results clearly demonstrate the positive effect of the core–shell structure on the capacitive performance. The superior capacitance of the ZnFe_2_O_4_@ZnCo_2_O_4_ electrode compared to conventional carbon materials is primarily attributed to the multi-electron redox chemistry of Co and Fe ions. The core–shell heterostructure provides a synergistic effect where the pseudocapacitive shell offers high charge storage capacity, exceeding the limits of traditional double-layer capacitance found in standard AC materials. Figure 7d shows the EIS Nyquist plots and equivalent circuit models for the three electrodes. The diameter of the semicircle in the high-frequency region corresponds to the charge transfer resistance (Rct), while the slope of the straight line in the low-frequency region reflects the ionic diffusion resistance. Comparison reveals that ZnFe_2_O_4_@ZnCo_2_O_4_ exhibits the lowest equivalent series resistance (Rs = 0.98 Ω) and charge transfer resistance (Rct = 1.26 Ω) among the three, while pure ZnFe_2_O_4_ shows Rs = 1.35 Ω and Rct = 2.14 Ω, and pure ZnCo_2_O_4_ shows Rs = 1.62 Ω and Rct = 2.87 Ω. The fitted EIS quantitative parameters directly confirm that the core–shell heterostructure effectively optimizes the material’s electronic conductivity and reduces interfacial charge transfer resistance. The superior electrochemical performance of the ZnFe_2_O_4_@ZnCo_2_O_4_ composite can be primarily attributed to the synergistic effect of its core–shell heterostructure. EIS results reveal that the ZnFe_2_O_4_@ZnCo_2_O_4_ electrode delivers a lower charge transfer resistance (Rct) than the single components, demonstrating that the heterojunction interface accelerates charge transfer kinetics. Meanwhile, the ZnFe_2_O_4_ nanorod substrate effectively inhibits the structural collapse and aggregation of ZnCo_2_O_4_ nanosheets during cycling. This structural arrangement not only ensures high mechanical stability but also maximizes the exposure of active sites, thereby significantly reducing the ion diffusion resistance and enhancing the overall capacitive performance.

**Figure 8:**
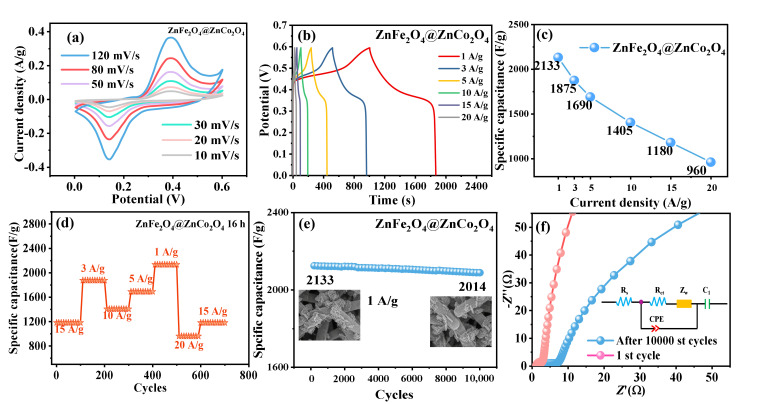
Electrochemical characterization of the ZnFe_2_O_4_@ZnCo_2_O_4_ electrode: (**a**) CV curves at different scan rates; (**b**) GCD curves at different current densities; (**c**) rate performance curves; (**d**) Step charge–discharge curves at varying current densities; (**e**) Long-term cycling stability at a current density of 1 A/g; (**f**) Comparison of Nyquist impedance curves before and after cycling.

To comprehensively evaluate the electrochemical performance of the ZnFe_2_O_4_@ZnCo_2_O_4_ electrode, we performed cyclic voltammetry (CV) at different scan rates, galvanostatic charge–discharge (GCD) tests at various current densities, rate-performance tests, and long-term cycling-stability tests. The results are shown in Figure 8. Figure 8a shows CV curves at different scan rates (10–120 mV/s). All curves exhibit a pair of distinct oxidation and reduction peaks, indicating the material’s typical pseudocapacitive energy storage mechanism. With increasing scan rate, the oxidation and reduction peaks shift positively and negatively, respectively, suggesting moderate polarization. The CV curves remain well-defined without obvious distortion, demonstrating favorable reaction kinetics and structural reversibility of the electrode. Figure 8b presents GCD profiles measured at current densities ranging from 1 to 20 A/g. All curves show nearly symmetric charge–discharge platforms, further verifying the highly reversible pseudocapacitive characteristics. As shown in the rate performance in Figure 8c, when the current density is increased from 1 A/g to 20 A/g, the specific capacitance of the electrode decreases from 2133 F/g to 960 F/g, with a capacity retention rate of approximately 45.0%, demonstrating good rate performance. This is attributed to the abundant mesoporous channels and low charge transfer resistance within the core–shell structure, which effectively facilitates efficient ion diffusion under high current densities. To further verify the material’s rate reversibility, we conducted step-charge/discharge tests at varying current densities (Figure 8d). At current densities of 15, 10, 5, 3, and 1 A/g, the electrode responded rapidly and recovered its corresponding specific capacitance, once again demonstrating its excellent rate reversibility and structural stability. The long-term cycling performance shown in Figure 8e demonstrates that the electrode retains a specific capacitance of 2014 F/g after 10,000 charge–discharge cycles at a current density of 1 A/g. The capacitance retention reaches 94.4%, indicating excellent cycling stability. To further verify the structural stability of the core–shell heterojunction during cycling, we compared the SEM images of the electrode material before and after the 10,000 cycles, which are also presented in Figure 8e. As shown in the SEM images, the one-dimensional rod-like morphology of the ZnFe_2_O_4_@ZnCo_2_O_4_ composite is well maintained after long-term cycling, and the core–shell hierarchical structure remains clearly intact without obvious collapse, pulverization, or peeling of the shell layer. Morphological characterization after long-term cycling directly confirms that the core–shell heterostructure effectively alleviates volume expansion during repeated charge–discharge cycles, accounting for the superior cycling stability of the electrode.

Furthermore, a comparison of the electrochemical impedance spectra before and after cycling (Figure 8f) reveals that the charge-transfer resistance of the electrode does not show significant changes after 10,000 cycles. This further confirms the excellent structural and interfacial stability of the electrode and provides a reliable basis for its practical application in supercapacitors.

**Figure 9:**
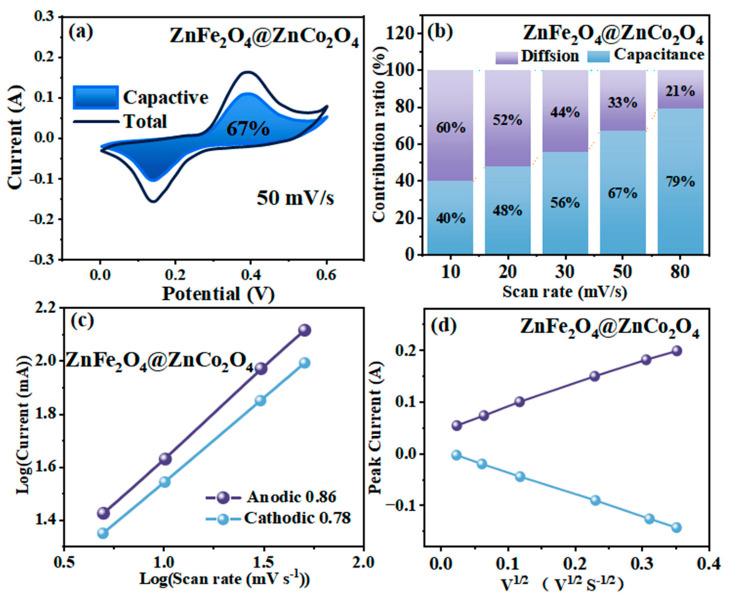
Analysis of charge storage kinetics in ZnFe_2_O_4_@ZnCo_2_O_4_ electrodes: (**a**) Decomposition of capacitive contributions at a scan rate of 50 mV/s; (**b**) Proportions of capacitive and diffusion-controlled contributions at different scan rates; (**c**) Log (i)–log (v) relationship between redox peak current and scan rate; (**d**) Relationship curve between peak current and the square root of the scan rate (v^1/2^).

To gain a deeper understanding of the charge storage kinetics in the ZnFe_2_O_4_@ZnCo_2_O_4_ electrode, we performed a quantitative kinetic analysis to decouple its capacitive contribution behavior based on cyclic voltammetry (CV) curves obtained at different scan rates. The results are shown in Figure 9. Figure 9a shows the capacitive contribution separation results at a scan rate of 50 mV/s, where the blue region represents the surface-controlled capacitive contribution, accounting for 67% of the total current. This indicates that, at this scan rate, the surface capacitive process has become the dominant energy storage mechanism. Figure 9b further summarizes the proportions of capacitive and diffusion-controlled contributions at different scan rates (10–80 mV/s). As the scan rate increases, the proportion of capacitive contribution gradually rises from 40% to 79%, while the diffusion-controlled contribution correspondingly decreases. This trend indicates that the contribution of ion diffusion processes is predominant at low scan rates, whereas as the scan rate increases, ion diffusion becomes limited, and surface capacitive processes gradually dominate. This is closely related to the high specific surface area and abundant active sites provided by the core–shell structure.

To further explore the reaction kinetics, linear fitting of log(i) versus log(v) was performed based on redox peak currents and scan rates (Figure 9c). The calculated b-values for anodic and cathodic peaks are 0.86 and 0.78, respectively, both ranging from 0.5 to 1.0. This indicates that the energy storage process of the electrode is simultaneously controlled by surface capacitance and ion diffusion, and exhibits distinct pseudocapacitive characteristics overall. Furthermore, based on the linear relationship between the peak current and the square root of the scan rate (v^1/2^) shown in Figure 9d, the anodic peak current exhibits a positive correlation with v^1/2^, while the cathodic peak current shows a negative correlation with v^1/2^. These results further confirm the existence of a typical diffusion-controlled process in the electrode. In summary, this synergistic “diffusion + capacitance” charge-storage mechanism, supported by the rapid ion/electron transport pathways constructed by the core–shell hierarchical structure, enables the electrode to achieve efficient charge storage over a wide range of scan rates, thereby providing a kinetic-level theoretical basis for its excellent rate performance.

**Figure 10:**
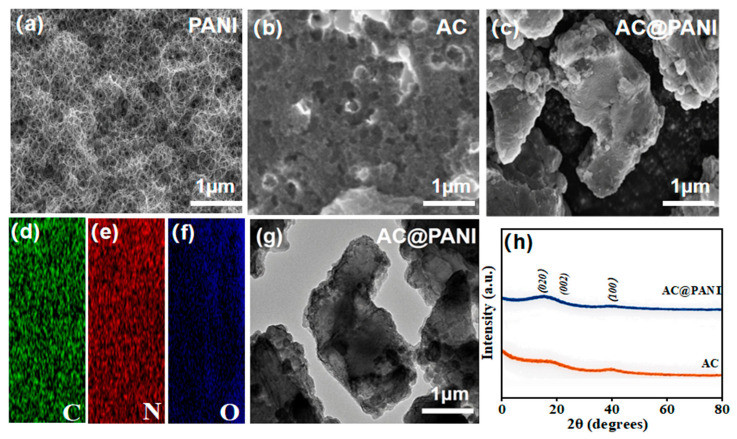
Characterization of the microstructure and elemental distribution of the anode materials: (**a**) SEM images of PANI, (**b**) AC, and (**c**) AC@PANI; (**d**–**f**) EDS elemental mapping of AC@PANI (C, O, N); (**g**) TEM image of AC@PANI; (**h**) XRD patterns of AC and AC@PANI.

To investigate the microstructure and elemental distribution of the anode materials, we characterized polyaniline (PANI), activated carbon (AC), and the activated carbon@polyaniline (AC@PANI) composite using scanning electron microscopy (SEM) and energy-dispersive X-ray spectroscopy (EDS). The results are shown in Figure 10. Figure 10a shows the SEM image of pure PANI. The sample exhibits a typical interwoven nanofiber network structure, with fibers interconnected to form a porous framework. This highly porous structure provides abundant pathways for ion transport; however, pure PANI is susceptible to agglomeration and volume expansion, which can lead to structural collapse during long-term cycling. In contrast, the SEM image of pure AC in Figure 10b reveals a rough surface with a distribution of numerous mesoporous and microporous structures. While this porous structure provides ample space for electrolyte penetration, the capacitive contribution of the AC material relies primarily on double-layer energy storage, restricting its potential for enhancing energy density. Figure 10c shows the SEM image of the AC@PANI composite material. It can be seen that PANI nanofibers uniformly coat the surface of AC particles, forming a “scaffold-coating” composite structure. This structure retains the porous scaffold characteristics of AC while improving the material’s conductivity and the exposure of pseudocapacitive active sites through the PANI coating, and simultaneously alleviates the agglomeration issues inherent to PANI. Further EDS elemental mapping analysis (Figure 10d–f) confirms the uniform distribution of C, O, and N elements in the AC@PANI composite. The C element mainly originates from the AC substrate, while O corresponds to the oxygen-containing functional groups on the AC surface and oxygen impurities in PANI. In addition, N is uniformly distributed over the entire material surface, confirming the uniform coating of PANI on the AC surface and providing abundant nitrogen-doped active sites for the composite. This synergistic composite structure of a porous AC scaffold and PANI nanofibers retains the high specific surface area and rapid ion transport pathways of the AC material while introducing the pseudocapacitive properties of PANI. At the same time, it effectively buffers the volume changes in PANI during charging and discharging, providing a structural foundation for enhancing the specific capacitance and cycling stability of the anode material. The TEM image of AC@PANI in Figure 10g further confirms that the AC particles are uniformly wrapped by PANI, showing a clear core–shell morphology without obvious phase separation, which is consistent with the SEM observations. The crystalline structures of AC and AC@PANI were analyzed by XRD, as shown in Figure 10h. Two broad diffraction peaks located at approximately 24° and 43° for pure AC are assigned to the (002) and (100) planes of amorphous carbon, respectively. Apart from the characteristic signals of AC, an additional weak peak near 20° corresponding to the (020) plane of PANI is detected for the AC@PANI composite, confirming the successful introduction of PANI. The broad and weak peaks suggest the low crystallinity of both materials, which is typical for amorphous carbon and polymer materials.

**Figure 11:**
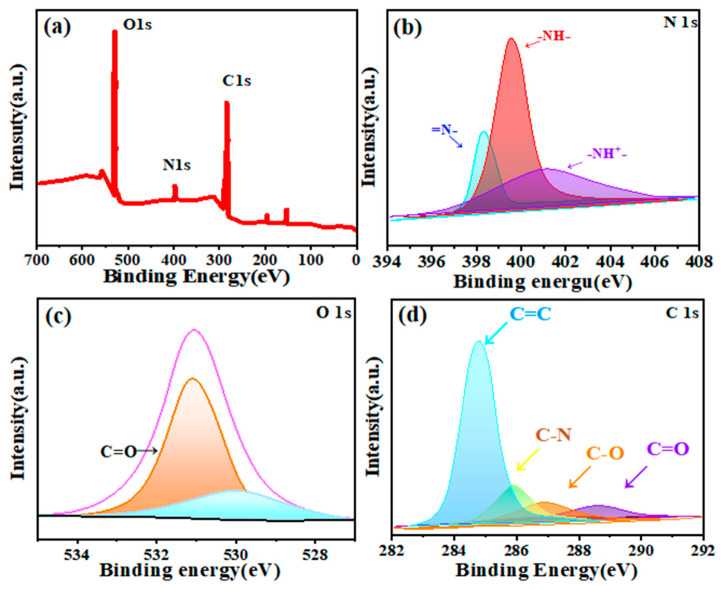
XPS characterization of the AC@PANI composite material: (**a**) Full XPS spectrum; (**b**) High-resolution N 1s spectrum; (**c**) High-resolution O 1s spectrum; (**d**) High-resolution C 1s spectrum.

To further elucidate the surface chemical composition and valence states of the AC@PANI anode material, we performed X-ray photoelectron spectroscopy (XPS) analysis, and the results are shown in Figure 11. The XPS survey spectrum in Figure 11a clearly shows three characteristic peaks at C 1s, O 1s, and N 1s, confirming the coexistence of C, O, and N elements in the composite material, which is consistent with the EDS elemental mapping results. The high-resolution N 1s spectrum (Figure 11b) can be deconvoluted into three distinct peaks: the =N^−^ peak at ~398.2 eV corresponds to imine nitrogen in the quinone structure, the -NH^−^peak at ~399.5 eV corresponds to secondary amine nitrogen in the benzene structure, and the -NH^+^- peak at ~401.2 eV corresponds to the protonated nitrogen species. Together, these three peaks confirm the successful polymerization of polyaniline. The presence of protonated nitrogen effectively enhances the material’s electronic conductivity and surface hydrophilicity, providing favorable conditions for the adsorption and transport of electrolyte ions. The O 1s spectrum in Figure 11c can be fitted with two characteristic peaks corresponding to the C=O bond at ~531.2 eV and the C-O bond at ~532.5 eV. These oxygen-containing functional groups mainly originate from the activated carbon substrate and the polyaniline surface. They not only provide additional pseudocapacitive contributions through redox reactions but also enhance the material’s surface wettability, promoting electrolyte penetration. The C 1s spectrum in Figure 11d can be deconvoluted into four characteristic peaks. The peak at ~284.6 eV corresponding to the C=C bond is attributed to the graphitic carbon backbone of the carbon material. The C–N bond at ~285.6 eV confirms the successful incorporation of nitrogen into the carbon framework. In addition, the C–O peak at ~286.5 eV and the C=O peak at ~288.8 eV are assigned to surface oxygen-containing functional groups, which are consistent with the results of the O 1s spectrum analysis. In summary, the XPS results confirm the successful loading of PANI and the realization of nitrogen doping in the AC@PANI composite, while also indicating that the material surface contains abundant oxygen- and nitrogen-containing functional groups. These functional groups offer extra pseudocapacitive active sites and improve the conductivity and hydrophilicity of the material, laying a chemical foundation for the enhancement of specific capacitance and rate performance of the anode.

**Figure 12:**
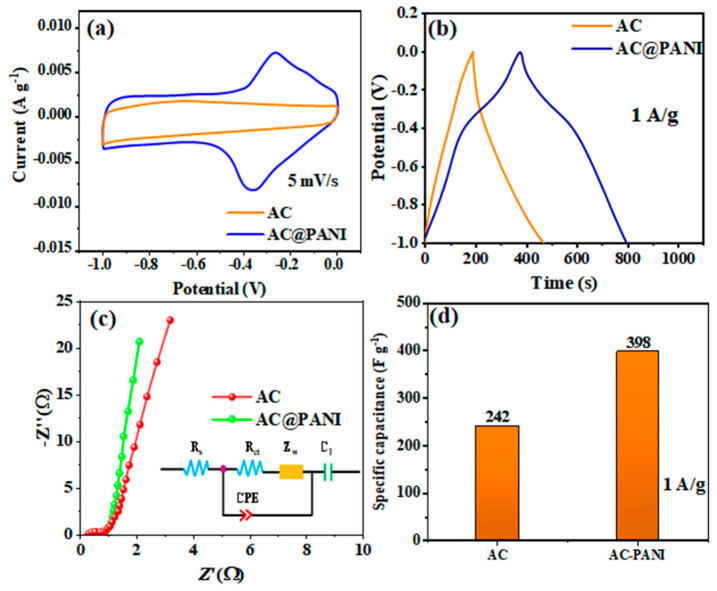
Comparison of electrochemical performance of the anode materials: (**a**) CV curves of AC and AC@PANI at a scan rate of 5 mV/s; (**b**) GCD curves at a current density of 1 A/g; (**c**) Electrochemical impedance spectroscopy (Nyquist curves) and equivalent circuits; (**d**) Comparison of specific capacitance at 1 A/g.

Figure 12a shows the CV curves of the two electrodes at a scan rate of 5 mV/s. The curve for pure AC exhibits a quasi-rectangular shape, which is typical of double-layer-capacitance-dominated behavior. In contrast, the CV curve of the AC@PANI electrode shows a much larger integrated area and distinct redox peaks. This indicates that the introduction of polyaniline has successfully introduced additional pseudocapacitive contributions to the composite system, overcoming the limitation of AC’s pure electrical double-layer capacitance (EDLC). This indicates a synergistic energy-storage mechanism involving both double-layer capacitance and pseudocapacitance, which substantially enhances the total charge-storage capacity of the electrode. At the same time, the AC@PANI curve retains good symmetry, indicating that the PANI coating has not impeded the ion transport kinetics of the AC substrate, and the electrode as a whole exhibits excellent reaction reversibility. Figure 12b displays GCD curves collected at 1 A/g. Both electrodes present well-symmetric charge–discharge profiles, implying high reversibility of the energy storage process. The AC@PANI electrode exhibits a longer discharge duration than pure AC, revealing its superior charge storage capability. Figure 12c shows the EIS Nyquist plots and equivalent circuit models for the two electrodes: the semicircular diameter in the high-frequency region corresponds to the charge transfer resistance (Rct), while the slope of the straight line in the low-frequency region reflects the ionic diffusion resistance. A comparison reveals that both the equivalent series resistance (Rs = 0.85 Ω) and the charge transfer resistance (Rct = 1.13 Ω) of the AC@PANI electrode are significantly lower than those of the pure AC electrode (Rs = 1.21 Ω, Rct = 1.96 Ω). These fitted EIS values indicate that the PANI coating improves electronic conductivity, enhances electrolyte wetting at the electrode interface, and reduces interfacial charge-transfer resistance, thereby supporting favorable electrochemical kinetics under high current densities.

Combined with the specific capacitance bar chart in Figure 12d, it can be seen that at a current density of 1 A/g, the specific capacitance of the AC@PANI electrode reaches as high as 398 F/g, an increase of approximately 64.5% compared to the 242 F/g of pure AC. Such prominent performance enhancement originates from the synergistic effect of two aspects. The redox reactions of PANI supply sufficient pseudocapacitive active sites, while the porous framework of AC acts as a supporting substrate for uniform PANI immobilization. This structure suppresses PANI aggregation and maintains the fast ion transport property of AC. While pure AC typically exhibits stable EDLC behavior, its specific capacitance is limited by its surface area. The introduction of PANI through in situ polymerization significantly enhances the total capacitance. This is because PANI contributes substantial pseudocapacitance through faradaic redox reactions, while the AC framework ensures high conductivity and maintains the structural integrity of the composite electrode.

**Figure 13:**
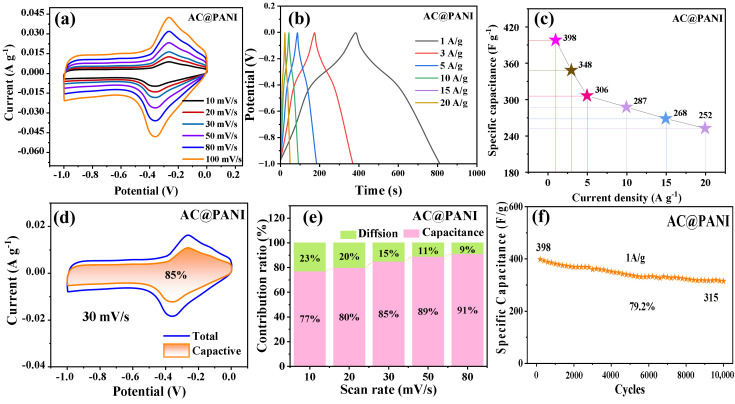
Electrochemical Performance and Kinetic Analysis of the AC@PANI Anode Material: (**a**) CV curves at different scanning rates; (**b**) GCD curves at different current densities; (**c**) Rate-dependent performance curves; (**d**) Capacitance contribution separation at 30 mV/s; (**e**) Proportion of capacitive versus diffusion-controlled contributions at different scan rates; (**f**) Long-cycle stability at a current density of 1 A/g.

To systematically investigate the electrochemical kinetics and energy storage mechanisms of the AC@PANI anode material, we conducted cyclic voltammetry (CV), rate performance, and long-term cycling stability tests at different scan rates. The results are shown in Figure 13. Figure 13a shows the CV curves of AC@PANI at scan rates ranging from 10 to 100 mV/s. All curves exhibit good symmetry, and the curve morphology is well-preserved as the scan rate increases, indicating that the electrode possesses excellent structural stability and fast response kinetics. The well-defined redox peaks confirm that the pseudocapacitive processes contributed by PANI operate synergistically with the double-layer capacitive behavior of the AC substrate during charge storage. Figure 13b shows the GCD curves at different current densities (1–20 A/g); all curves exhibit approximately symmetrical charge–discharge characteristics, further validating the high reversibility of the electrode. As shown in the rate-performance curve in Figure 13c, when the current density increases from 1 A/g to 20 A/g, the specific capacitance decreases from 398 F/g to 252 F/g, corresponding to a retention rate of 63.3% and demonstrating good rate capability. This superior performance stems from the rapid ion transport pathways provided by the porous AC scaffold and the high conductivity resulting from the uniform PANI coating, which effectively ensures charge transfer efficiency under high current densities.

To further elucidate the charge storage kinetics, we performed a quantitative analysis of capacitance contributions in CV curves at different scan rates. Figure 13d shows the capacitance contribution decomposition at a scan rate of 30 mV/s, where the surface-controlled capacitance contribution accounts for as much as 85%, indicating that at this scan rate, double-layer capacitance and surface pseudocapacitance processes have become dominant. Figure 13e summarizes the capacitive and diffusion-controlled contributions at various scan rates. As the scan rate increases from 10 mV/s to 80 mV/s, the capacitive contribution gradually increases from 77% to 91%, accompanied by a decline in the diffusion-controlled contribution. This trend indicates that at low scan rates, the ion diffusion process contributes more significantly to the total capacitance. In contrast, as the scan rate increases and ion diffusion becomes restricted, surface capacitance processes gradually dominate. This behavior is closely related to the porous structure and high specific surface area of the electrode material. Long-term cycling stability tests (Figure 13f) show that after 10,000 charge–discharge cycles at a current density of 1 A/g, the specific capacitance of the electrode remained at 315 F/g, with a capacity retention rate as high as 79.2%. This indicates that the support provided by the AC scaffold effectively suppressed the volume expansion and structural collapse of PANI during the charge–discharge process, endowing the electrode with excellent cycling stability.

**Figure 14:**
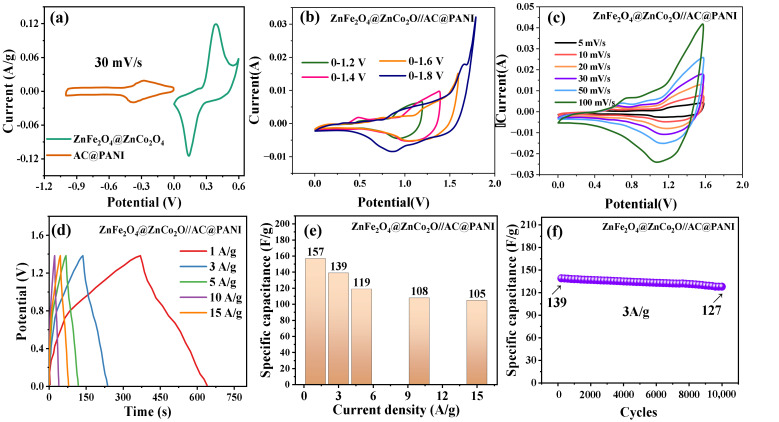
Electrochemical performance of the ZnFe_2_O_4_@ZnCo_2_O_4_//AC@PANI supercapacitor: (**a**) CV curves of the positive and negative electrodes at 30 mV/s; (**b**) CV curves at different voltage windows; (**c**) CV curves at different scanning rates; (**d**) GCD curves at different current densities; (**e**) Rate performance curves; (**f**) Long-cycle stability at a current density of 3 A/g.

To comprehensively evaluate the overall electrochemical performance of the ZnFe_2_O_4_@ZnCo_2_O_4_//AC@PANI hybrid supercapacitor (HSC) device, we systematically characterized its voltage window, rate capability, and cycling stability. The results are shown in Figure 14. Figure 14a presents the CV curves of the anode and cathode materials at a scan rate of 30 mV/s. The ZnFe_2_O_4_@ZnCo_2_O_4_ cathode exhibits an operating potential window of 0–0.6 V and shows typical pseudocapacitive redox behavior. In contrast, the AC@PANI anode operates in a potential window of −1.0 to 0 V and demonstrates synergistic charge-storage behavior involving both electric double-layer capacitance and surface pseudocapacitance. The complementary potential characteristics of the two materials lay the foundation for constructing a wide voltage window in the device. Figure 14b shows the CV curves of the device under different voltage windows (0–1.2 V to 0–1.8 V). As the voltage window is expanded to 1.6 V, the curve profile remains stable without obvious polarization or side reactions related to oxygen evolution. When the voltage window is further increased to 1.8 V, significant polarization and current distortion appear, indicating that 0–1.6 V is the optimal stable operating window for the device. This wide voltage window is a key prerequisite for improving the energy density of the device. Figure 14c presents CV curves of the device at scan rates from 5 to 100 mV/s. No obvious distortion is observed with increasing scan rate, and the redox peak current rises linearly, indicating outstanding rate capability. This indicates that the device can maintain stable charge storage behavior during rapid charging and discharging, which is attributed to the synergistically optimized ion/electron transport kinetics of the anode and cathode materials. Figure 14d shows the GCD curves at different current densities (1–15 A/g); all curves exhibit approximately symmetrical charge–discharge characteristics, confirming the high reversibility of the device. Combined with the specific capacitance data in Figure 14e, the device delivers a specific capacitance of 157 F/g at 1 A/g. When the current density is increased to 15 A/g, it still maintains a specific capacitance of 105 F/g, corresponding to a capacity retention of approximately 66.9%. This demonstrates excellent rate performance, which is attributed to the good matching and synergistic enhancement between the anode and cathode materials. Long-term cycling stability tests (Figure 14f) show that after 10,000 charge–discharge cycles at a current density of 3 A/g, the specific capacitance of the device decreases from an initial 139 F/g to 127 F/g, with a capacity retention of 91.4%. This indicates that the hybrid supercapacitor exhibits excellent structural and electrochemical stability.

The pseudocapacitive behavior of the electrodes was further analyzed from a mechanistic perspective. For the ZnFe_2_O_4_@ZnCo_2_O_4_ positive electrode, the charge storage mechanism is primarily governed by the fast and reversible Faradaic redox reactions occurring at or near the surface of the hierarchical core–shell structure. Specifically, in the acidic electrolyte, the hydronium ions (H_3_O^+^) interact with the mixed-valence metal centers (Zn, Fe, and Co), facilitating the transition between different oxidation states of the transition metal oxides. The ZnFe_2_O_4_ rod-like core provides a stable framework for electron conduction, while the vertically grown ZnCo_2_O_4_ nanosheet shell offers abundant active sites for proton-coupled electron transfer, thereby enhancing redox kinetics. Similarly, for the AC@PANI negative electrode, the pseudocapacitance is attributed to the synergistic effect between the double-layer capacitance of the activated carbon and the Faradaic activity of the polyaniline (PANI). In acidic media, PANI undergoes continuous protonation and deprotonation, leading to a transition between its different oxidation states. This reversible doping/dedoping process involving acid anions allows for high charge storage capacity and rapid charge–discharge response.

**Figure 15:**
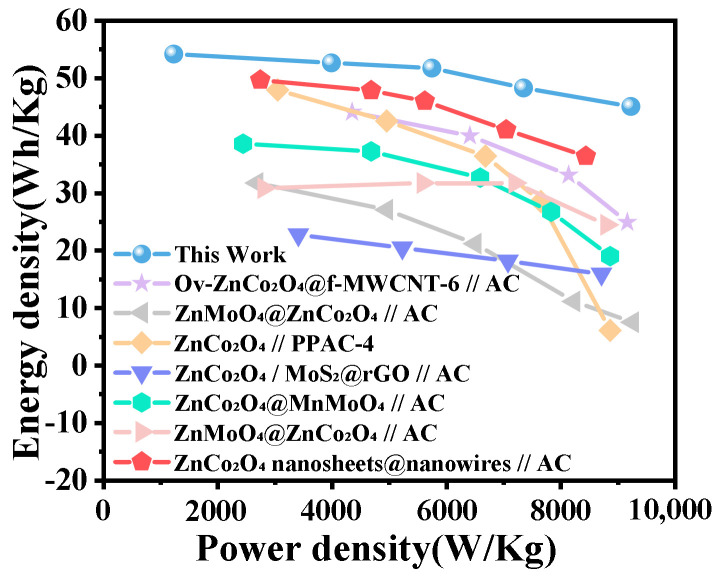
Comparison of Ragone curves for the ZnFe_2_O_4_@ZnCo_2_O_4_//AC@PANI supercapacitor device fabricated by this working group.

To evaluate the practical application potential of the ZnFe_2_O_4_@ZnCo_2_O_4_//AC@PANI hybrid supercapacitor (HSC) assembled in this work, a Ragone plot of energy density versus power density was constructed and compared with recently reported ZnCo_2_O_4_-based supercapacitor systems. The results are shown in Figure 15. As shown in the Ragone plots, the device constructed in this work exhibits excellent synergy between energy density and power density. At a power density of 1600 W/kg, the maximum energy density reaches 54.7 Wh/kg. Even at a high power density of 9000 W/kg, it still maintains an energy density of 45.2 Wh/kg, significantly outperforming other comparison systems, such as ZnCo_2_O_4_ nanosheets@nanowires//AC and ZnCo_2_O_4_@MnMoO_4_//AC [29,41,42,43,44,45,46]. This outstanding electrochemical performance is mainly attributed to several factors. First, the ZnFe_2_O_4_@ZnCo_2_O_4_ core–shell heterostructure used as the cathode provides abundant redox-active sites and rapid ion/electron transport pathways, thereby endowing the electrode with high specific capacitance. Second, the AC@PANI composite anode achieves the synergistic enhancement of electric double-layer capacitance and pseudocapacitance, effectively improving the charge-storage capability of the anode. More importantly, the precise matching between the cathode and anode materials enables the device to operate stably within a wide voltage window of 1.6 V, which is one of the key factors contributing to the enhanced energy density. Compared with previously reported devices of the same type, the HSC device in this study demonstrates significant advantages in both energy density and power density. These results suggest that the combination of ZnFe_2_O_4_@ZnCo_2_O_4_ and AC@PANI has strong potential for constructing high-performance energy-storage devices and provides a feasible reference for the design and optimization of future hybrid supercapacitors.

To bridge the gap between laboratory-scale research and the high-power demands of new energy electric vehicles (EVs), several practical factors must be considered. Although the current study utilizes CR2032 coin cells for material performance validation, the high specific capacitance (2133 F/g) and energy density (54.7 Wh/kg) of the ZnFe_2_O_4_@ZnCo_2_O_4_//AC@PANI system demonstrate significant potential for EV energy storage. Regarding safety, the use of an aqueous electrolyte inherently eliminates the risk of thermal runaway and fire, which is a critical concern for EV batteries. Multiple cells can be connected in series to elevate the operating voltage for matching the high-voltage bus of electric drive systems. For practical electric vehicle applications, follow-up research will focus on fabricating pouch cells with these materials to reduce packaging weight and optimize heat dissipation under high-rate charge–discharge conditions.

## 4. Conclusions

In this work, ZnFe_2_O_4_@ZnCo_2_O_4_ core–shell cathode materials and AC@PANI composite anode materials were designed and prepared for hybrid supercapacitors. The electrodes were fabricated by conventional slurry coating onto nickel foam. The core–shell structure provides stable mechanical support, increases specific surface area, enriches active sites, and reduces charge transfer resistance. The cathode delivers 2133 F/g at 1 A/g with 94.4% retention after 10,000 cycles. The AC@PANI anode achieves 398 F/g at 1 A/g with good cycling stability. The assembled ZnFe_2_O_4_@ZnCo_2_O_4_//AC@PANI hybrid supercapacitor shows a voltage window of 0–1.6 V, a high energy density of 54.7 Wh/kg at 1600 W/kg, and 91.4% capacity retention after 10,000 cycles. This performance is superior to most reported ZnCo_2_O_4_-based systems. This study provides a feasible strategy for constructing high-performance core–shell electrodes and hybrid supercapacitors and may support the development of energy-storage devices for new energy electric vehicles.

## Figures and Tables

**Table 1:** Microstructural parameters of pure ZnFe_2_O_4_ and ZnFe_2_O_4_@ZnCo_2_O_4_ composite calculated from XRD data.

Sample	Lattice Parameter a (Å)	Crystallite Size D (nm)	Microstrain ε (×10^−3^)	Dislocation Density δ (×10^14^ Line/m^2^)
ZnFe_2_O_4_	8.441	28.5	1.22	12.3
ZnFe_2_O_4_@ZnCo_2_O_4_	8.435	32.1	1.45	9.7

## Data Availability

The original contributions presented in this study are included in the article. Further inquiries can be directed to the corresponding author.
